# Isolated Plexiform Neurofibroma at the Liver Hilum in a Pediatric Patient: A Case Report

**DOI:** 10.7759/cureus.113732

**Published:** 2026-07-31

**Authors:** Prashant Obed R Dundi, Sahith Kaki, Andrew E Toader, Nadia Asif

**Affiliations:** 1 Family Medicine, Allegheny Health Network, Erie, USA; 2 Medicine, Lake Erie College of Osteopathic Medicine, Erie, USA; 3 Pediatrics, Allegheny Health Network, Erie, USA

**Keywords:** hepatic hilum, liver neurofibroma, neurofibromatosis type 1 (nf1), pediatric, plexiform neurofibromas

## Abstract

Neurofibromatosis type 1 (NF1) is a common autosomal dominant genetic disorder, presenting with hallmark benign tumors including cutaneous and plexiform neurofibromas. Plexiform-type neurofibromas most commonly present in peripheral tissue nerves underlying the dorsal trunk, but rarely present in visceral organs such as the liver. This case presents a 12-year-old male patient with a history of NF1 who presented to the emergency department with non-specific abdominal pain. Hepatic sonography showed an infiltrating, nodular enhancement along the porta hepatis. A diagnosis of plexiform neurofibroma was made via MRI of the liver. Plexiform neurofibromas of the liver are a rare finding in patients with diagnosed NF1. However, plexiform neurofibromas should be considered as part of the differential diagnosis in NF1 patients presenting with a hepatic mass due to rare, but possible, malignant transformation. The potential transformation to malignant peripheral nerve sheath tumor (MPNST) requires post-diagnosis monitoring. This case adds to the body of literature highlighting a rare presentation of hepatic neurofibroma in a child with NF1, outlining the importance of considering plexiform neurofibroma as a differential diagnosis in an unknown hepatic or gastrointestinal visceral lesion.

## Introduction

Neurofibromatosis type 1 (NF1) is a common autosomal dominant genetic disorder characterized by the development of various benign and malignant tumors of the nervous system. Common benign tumors include cutaneous and plexiform neurofibromas and optic gliomas, while possible malignant tumors include high-grade gliomas, gastrointestinal stromal tumors, and neuroendocrine tumors.

Plexiform neurofibromas are highly suggestive of NF; however, their occurrence in the liver is rare, impacting ~30-50% of NF1 patients. Plexiform neurofibromas are primarily tumors of Schwann cells and most commonly occur in tissues on the dorsal trunk, on the extremities, and in the pelvis. Hepatic plexiform neurofibromas are reported in the literature in only a handful of children [1-5]. These lesions can mimic other hepatic masses, and misdiagnosis or delayed recognition carries management risks.

Because of their rarity and potential for misdiagnosis, hepatic plexiform neurofibromas pose a diagnostic challenge. We report such a case in a pediatric NF1 patient, who presented with a neurofibroma at the hepatic hilum. This case highlights the need for clinicians to consider hepatic involvement in NF1 patients presenting with upper abdominal masses. Hepatic plexiform neurofibromas are particularly underreported, making this case valuable to expand the differential diagnosis for portable hepatic lesions [1-5].

## Case presentation

A 12-year-old African-American male patient with a past medical history of hereditary genetic NF1 which was diagnosed by age 8, asthma, autism, and attention-deficit/hyperactivity disorder presented to the emergency department with vague abdominal pain and intermittent right upper quadrant abdominal pain for the past week. On detailed history, nausea, vomiting, diarrhea, constipation, fever, chills, flank pain, and dysuria were all denied. On review of systems, the patient endorsed vague abdominal pain and cramping. Vital signs during examination were stable and normal except for mildly elevated blood pressure. Physical exam was significant for positive Chvostek sign, café-au-lait macules on the upper chest, and neurofibromas on the upper head, anterior chest, abdomen, and upper back. Abdominal exam revealed a soft, non-tender abdomen with no pulsatile masses and no overlying ecchymosis. There were no signs of appendicitis, cholecystitis, or peritonitis. The findings of the initial blood work are presented in Table 1, with low serum calcium, low vitamin D level, elevated parathyroid hormone level, and indirect hyperbilirubinemia. Leukopenia, which was chronic for this patient, was also seen in the initial workup.

**Table 1:** Lab findings from the initial workup in the emergency department revealing hypocalcemia, on further evaluation consistent with secondary hyperparathyroidism. WBC: white blood cell count; PTH: parathyroid hormone; k: kilo; mcL: microliter; dL: deciliter; U: unit; mL: milliliter; pg: picogram; ng: nanogram; g: gram; mg: milligram

Test	Result	Reference
WBC (k/mcL)	3.13	4.50-13.50
Hemoglobin (g/dL)	14.7	13.0-16.0
Calcium (mg/dL)	6.1	9.0-11.5
Albumin (g/dL)	4.3	4.0-5.0
Alkaline phosphatase (U/L)	829	500-1000
Gamma-glutamyl transferase (U/L)	18	0-23
Direct bilirubin (mg/dL)	0.4	<0.3
Total bilirubin (mg/dL)	2.0	0.3-1.2
Aspartate aminotransferase (U/L)	20	10-50
Alanine aminotransferase (U/L)	8	5-55
PTH (pg/mL)	365.1	11.0-68
25-Hydroxy vitamin D (ng/mL)	6.7	30.0-100.0
Ionized calcium (mmol/L)	0.85	1.12-1.23

The patient was admitted to the acute pediatric inpatient unit. A further evaluation was done for hypocalcemia, which revealed an elevated parathyroid hormone level, a low 25-hydroxy vitamin D level, and a low ionized calcium level consistent with secondary hyperparathyroidism due to vitamin D deficiency. At this point, other causes of the patient's vitamin D deficiency like dietary issues and sunlight exposure were ruled out, since the patient had a well-balanced appetite with good calcium intake and enough sunlight exposure. Hence, he was started on a vitamin D3 supplement (Table 1).

**Figure 1:**
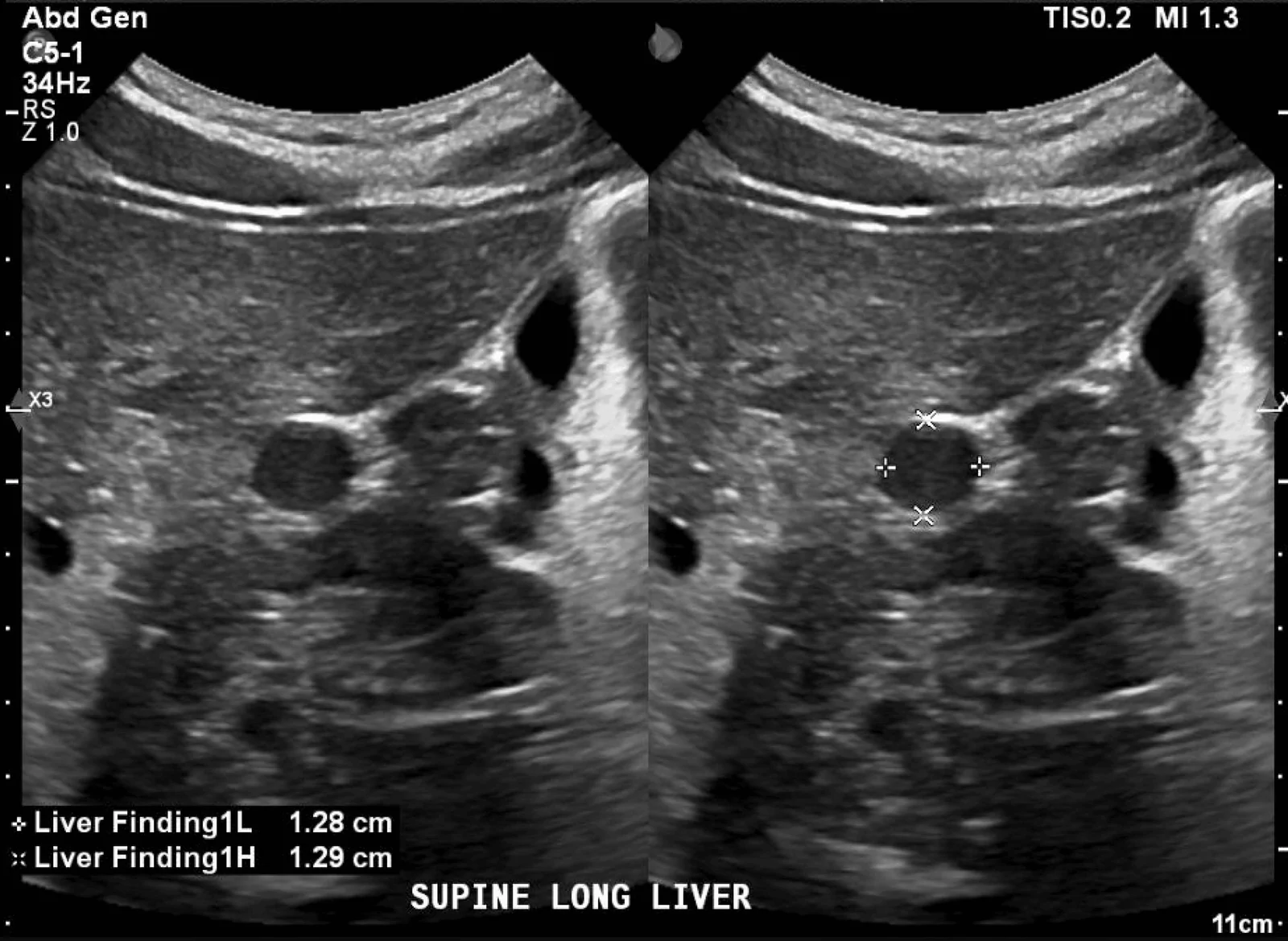
Liver ultrasound revealing a rounded, hypoechoic mass in the hepatic hilum measuring 13 × 13 × 18 mm (length and height). The liver echotexture is heterogeneous.

**Figure 2:**
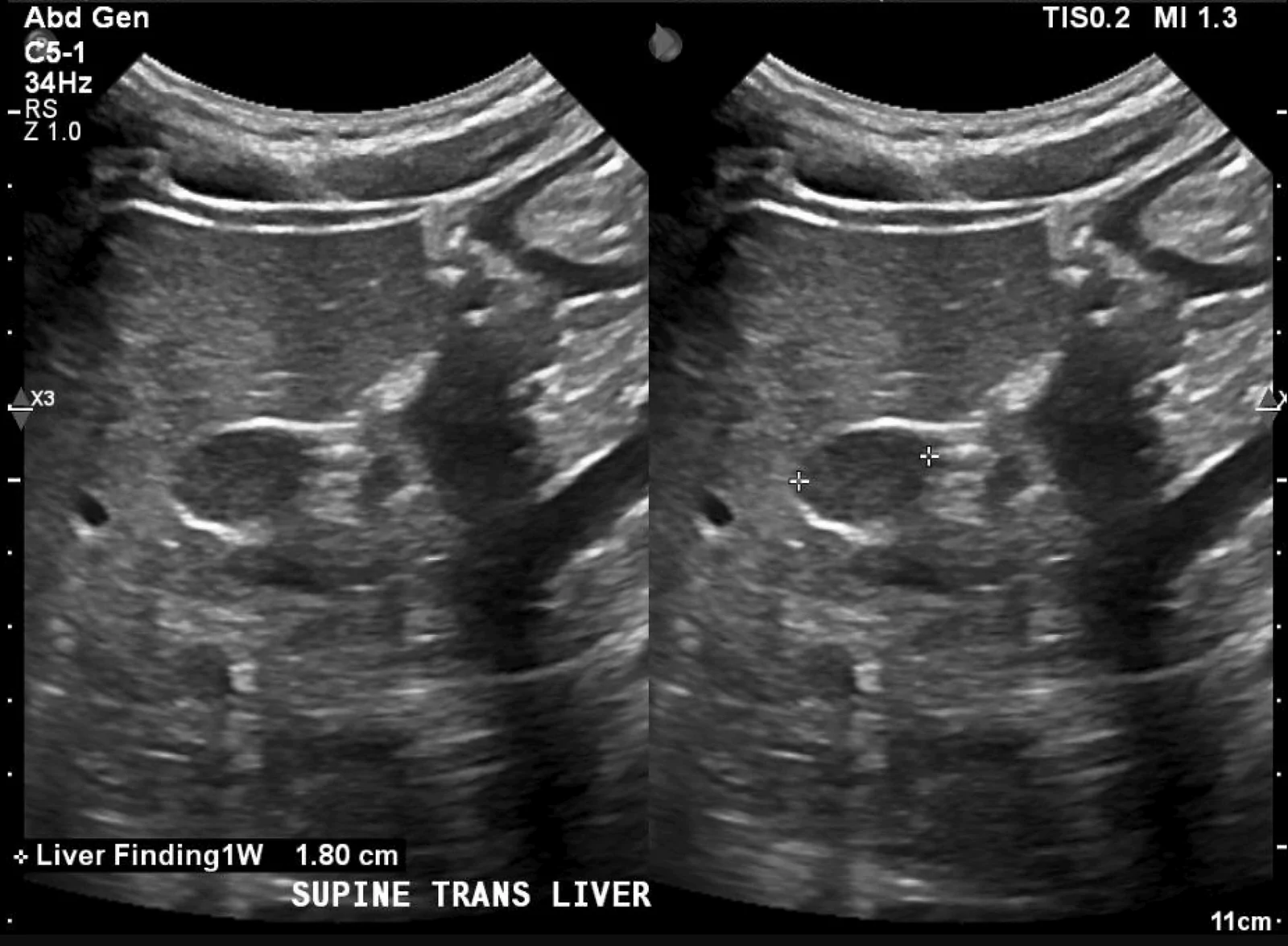
Liver ultrasound revealing a rounded, hypoechoic mass in the hepatic hilum measuring 13 × 13 × 18 mm (width). The liver echotexture is heterogeneous.

**Figure 3:**
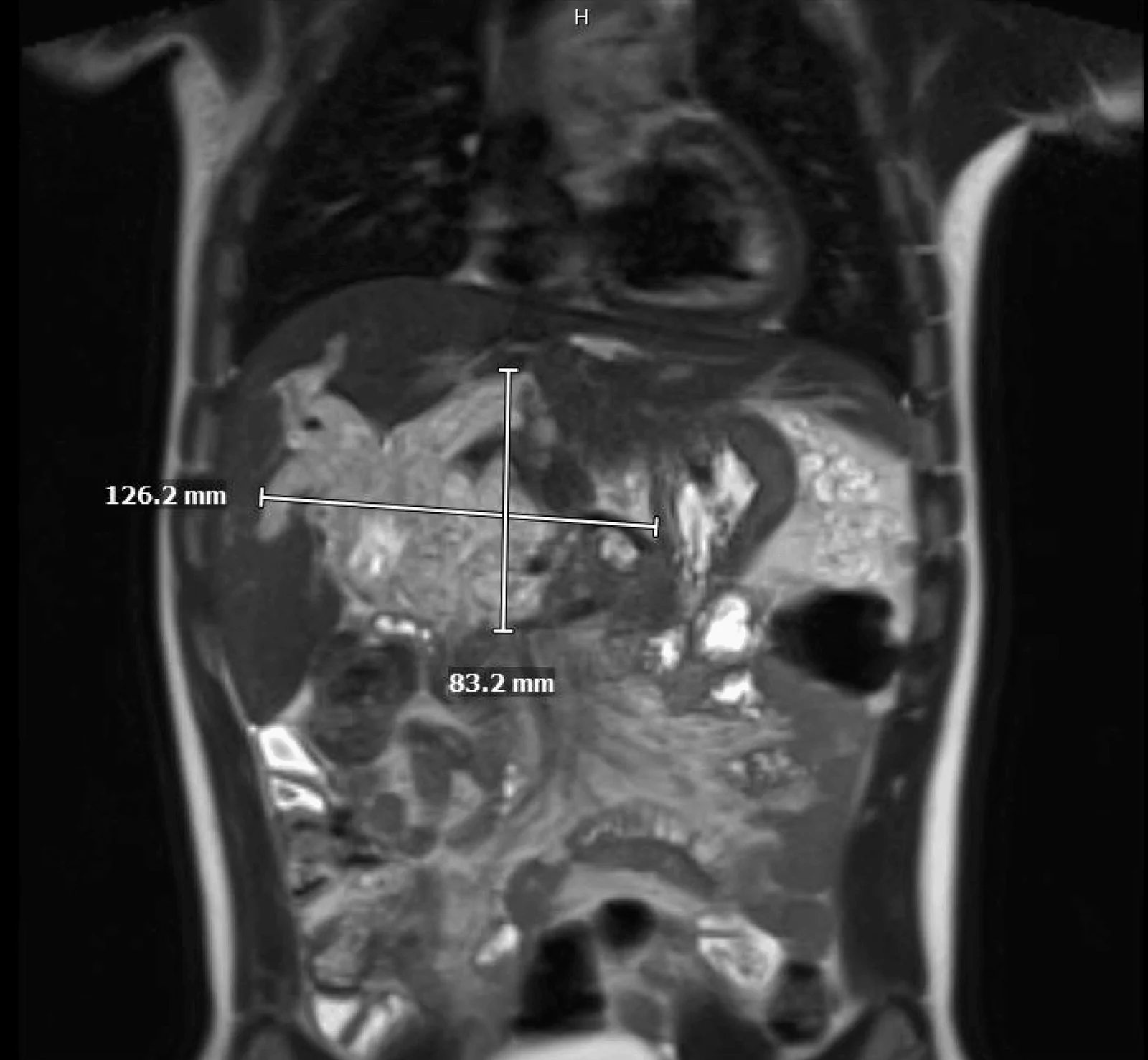
Liver MRI revealing a heterogeneously T2 hyperintense infiltrating mass-like lesion with somewhat tubular components. It is visualized centered in the porta hepatis surrounding the portal vasculature, extending into the liver along the portal vasculature, surrounding the proximal pancreas and celiac axis abutting the IVC, with some mild internal as well as nodular enhancement, suggesting a plexiform neurofibroma. This measures approximately 12.6 × 8.3 cm. IVC: inferior vena cava

Ultrasound of the liver revealed a rounded, hypoechoic mass in the hepatic hilum which measured 13 × 13 × 18 mm (Figure 1, Figure 2). MRI of the liver with and without contrast revealed a heterogeneous T2 hyperintense infiltrative lesion centered in the porta hepatis extending into the liver along the portal vasculature with some mild internal nodular enhancement, suggesting a plexiform neurofibroma (Figure 3). The tumor encased but did not obstruct the portal vein and mildly displaced the central right and left hepatic ducts, which were prominent; no further peripheral ductal dilation was seen (Figure 3). The patient was transferred to the regional quaternary care children's hospital after the treatment and resolution of acute hypocalcemia, and it was determined that the hepatic plexiform neurofibroma did not warrant urgent surgery due to its mild symptomatology. The patient's elevated bilirubin spontaneously resolved without treatment. He will continue to follow up with the specialized NF1 outpatient clinic routinely to monitor the progression of the disease.

## Discussion

NF1 is an autosomal dominant disorder caused by mutations in the NF1 gene on chromosome 17q11.2, producing neurofibromin, a tumor suppressor protein that negatively regulates the RAS signaling pathway. NF1 has an incidence of one in 3,000 and is characterized by increased risk for several benign and malignant tumors. The National Institutes of Health (NIH) specifies the diagnostic criteria of NF1 as requiring two of the following findings: six or more café-au-lait macules, freckling in the axillary or inguinal area, neurofibromas, optic gliomas, iris hamartomas or choroidal abnormalities, osseous lesions, or a first-degree relative diagnosed with NF1.

The hallmark sign of NF1 is the presence of neurofibromas, which are visualized on the skin or present throughout the peripheral nervous system. The body of existing literature shows that up to 30-50% of patients with NF1 have plexiform neurofibromas [6]. Visceral involvement in NF1 such as in the gastrointestinal tract and other abdominal organs is uncommon. Most deep neurofibromas are of the plexiform type and involve the retroperitoneal/paraspinal, sacral plexus, sciatic notch, and perirectal areas. There are few cases of isolated hepatic neurofibromas in the literature [2,3]. They are usually discovered incidentally or as part of a comprehensive NF1 workup [2,3].

A search of PubMed with the keywords ("liver" or "porta hepatis") and "neurofibroma" and "case" revealed 25 case reports of plexiform neurofibroma in the liver, of which six were pediatric patients [2,7-14]. Although the presence of a plexiform neurofibroma is highly suggestive of NF1 disease, one of the cases presented a male adult without NF1 disease (no family history nor any other diagnostic features present) [9]. Of the 25 cases, seven patients presented with a hepatic neurofibroma as the sole lesion related to NF1 [7,8,10-14]. Of the 25 cases, seven underwent surgical resection, mainly related to symptomatic presentation, and four had a biopsy performed due to uncertain diagnosis.

Plexiform hepatic neurofibromas may mimic other hepatic masses, and imaging findings are sometimes non-specific; hence, the gold standard method for diagnosing neurofibromas is histopathologic examination. On T1-weighted sequences, a hypointense signal can be seen, and on T2-weighted or other water-sensitive sequences, a hyperintensity with a central target can be appreciated [1]. Biopsy of the lesion is generally only recommended if the imaging findings are not diagnostic or if progression to a malignant lesion is suspected. Neurofibromas are composed of Schwann cells, fibroblasts, and perineurial cells, and 50% of them show positive immunohistochemistry for the S100 protein [6]. Care should be taken when evaluating these neurofibromas, as transformation to malignant peripheral nerve sheath tumor (MPNST), though rare in children, warrants close monitoring. In this patient, it was determined from the MRI findings and specialist consultation that this mass was an asymptomatic plexiform neurofibroma; thus, it did not warrant a biopsy or surgical resection. Serial imaging will be done of the lesion to monitor for growth or malignant transformation.

## Conclusions

This case demonstrates a rare presentation of an unknown liver mass in a patient with NF1, which was found to be a hepatic plexiform neurofibroma. Given the prevalence of NF1, atypical presentations with visceral neurofibromas should raise clinical suspicion for NF1-related disease, even in the absence of cutaneous findings. In this patient, follow-up included a hepatologist, an oncologist, a general surgeon, and a specialized NF1 clinic at the local quaternary care children's hospital. In conclusion, this case adds to the body of literature highlighting a rare presentation of hepatic neurofibroma in a child with NF1, outlining the importance of considering plexiform neurofibroma as a differential diagnosis in an unknown hepatic or gastrointestinal visceral lesion.
